# Animal models of cisplatin‐induced neuropathic pain

**DOI:** 10.1002/ame2.12548

**Published:** 2025-01-23

**Authors:** Ningxin Li, Mingzhu Li, Shengbo Jin, Jun Yu, Hongzhe Wei, Wenping Wang, Siyao Ma, Yuxin Jiang, Qian Liu, Huini Yao

**Affiliations:** ^1^ China Medical University Shenyang China; ^2^ Cancer Hospital of China Medical University, Liaoning Cancer Hospital & Institute Sheng Yang China; ^3^ Liaoning University of Traditional Chinese Medicine Shenyang China

**Keywords:** animal models, chemotherapy, cisplatin, peripheral neuropathic pain

## Abstract

Cisplatin chemotherapy has been used as the main treatment for different types of cancer. However, cisplatin chemotherapy‐induced peripheral neuropathic pain (CIPNP) seriously affects the treatment process and quality of life of patients. In addition, it impacts the underlying mechanism and prevention and treatment strategies, indicating that drug selection and efficacy evaluation need to be further investigated. Furthermore, an animal model that is more consistent with the pathological mechanism needs to be developed. In this study, we describe and discuss the methods of developing and detecting CIPNP models in rats and mice induced by cisplatin chemotherapy. The aim was to improve the modeling rate and develop animal models that are more consistent with the developmental pattern of the disease. In addition, the study provides ideal reference animal models for clinical research and drug discovery and development.

## Graphical abstract

This article illustrates the modeling process of a cisplatin‐induced neuropathic pain model in rats or mice. Multiple modeling tests are required for rats or mice before, during, and after modeling. The appropriate injection method, dose, and modeling method are selected, and multiple assays are chosen for evaluation and comparison.

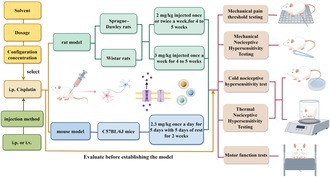

## 1 INTRODUCTION

Cisplatin is a first‐line cytotoxic antitumor drug used to treat different types of solid tumors, such as small‐cell and non‐small‐cell lung cancer, bladder cancer, and testicular cancer. However, more than a quarter of patients on this therapy experience neuropathic pain, primary clinical manifestations such as tingling, burning, sensation or numbness in the hands and feet, and diminished sense of touch. Moreover, the therapy has to be terminated in severe cases.^1^ The American Society of Clinical Oncology (ASCO) recommends the use of antidepressants, analgesics, neuroprotective drugs, antiepileptics, or hypothermic hand and foot sleeves/compression gloves for treatment in the Guidelines for the Prevention and Management of Chemotherapy‐Induced Peripheral Neuropathy in Adult Cancer Survivors.^2^ However, all these are only symptomatic in nature. In addition, their efficacy is poor, and the adverse effects of long‐term application are large. In contrast, Chinese medicines have significant efficacy in preventing and treating cisplatin chemotherapy‐induced peripheral neuropathic pain (CIPNP) and can improve the quality of life of patients after chemotherapy. Therefore, it is essential to develop ideal rat and mouse models of CIPNP and elucidate specific modeling detection methods, which can promote the prevention and treatment of CIPNP, the research and development of new drugs, and the improvement in patients' quality of life after chemotherapy. In this paper, we describe the modeling methods and modeling assays of CIPNP models in rats and mice. The aim was to improve the modeling rate, develop animal models that are more in line with the pathological mechanisms, and provide important references for further experimental and clinical studies.

## 2 ANIMAL SELECTION AND MODEL DEVELOPMENT METHOD

The effect of pain on rodents is remarkable. The rat and mouse CIPNP models are advantageous due to the ease of simulating injuries and performing operations, and a high rate of modeling; these have emerged as the preferred CIPNP models for researchers. The commonly used dose and regimen of cisplatin in clinics is 40–120 mg/m^2^, day 1, repeated every 3 weeks; or 15–20 mg/m^2^, days 1–5, repeated every 3–4 weeks. The neurotoxicity of cisplatin is dose dependent, with symptoms usually appearing in patients receiving cumulative doses of up to 250–500 mg/m^2^ and partially resolving in most patients after discontinuation of the drug.^3^ Therefore, CIPNP models in rats and mice are largely chronic models, and the injection modes are usually intraperitoneal injection and tail vein injection, which are relatively simple, easy to perform, and safe. However, currently, the development of CIPNP models in rats and mice is based on intraperitoneal injection, and the primary adverse reactions caused by intraperitoneal injection are peritoneal swelling and the production of ascites^4^ (Table 1).

**TABLE 1:** CIPNP model injection method of rat and mice.

Injection methods	Configuration concentration	Dosage mg/(kg·day)	Solvent
i.p.^1^, ^4^, ^5^, ^6^, ^7^, ^8^, ^9^, ^10^, ^11^, ^12^, ^13^, ^14^, ^15^, ^16^, ^17^, ^18^, ^19^, ^20^, ^21^, ^22^, ^23^, ^24^, ^25^, ^26^, ^27^, ^28^, ^29^, ^30^, ^31^, ^32^, ^33^, ^34^, ^35^, ^36^, ^37^, ^38^, ^39^, ^40^, ^41^, ^42^, ^43^	1 mg/mL	1	Normal saline
	0.08–0.15 mg/mL	2	Normal saline
	20 mg/mL	4	Normal saline
	0.2 mg/mL	2	0.4% Dimethyl sulfoxide
	2 mg/mL	2	0.4% Dimethyl sulfoxide
	0.2 mg/mL	2	Phosphate buffered saline
	0.23 mg/mL	2.3	Phosphate buffered saline
i.v.^43^, ^44^	Not mentioned	2	Normal saline
	Not mentioned	2.3	Normal saline

Abbreviations: CIPNP, chemotherapy‐induced peripheral neuropathic pain; i.p., intraperitoneal injection; i.v., intravenous injection.

### 2.1 Development of rat model

Researchers mostly simulate clinical multiple chemotherapies, selecting 2 mg/kg injected once or twice a week or 3 mg/kg injected once a week for 4–5 weeks to develop a chronic model of CIPNP^4^, ^5^, ^6^, ^7^, ^8^, ^9^, ^10^, ^11^, ^12^, ^13^, ^14^, ^15^, ^16^ (Table 2). When a single dose of 2 mg/kg of cisplatin was injected, with a cumulative dose of 6 mg/kg, and the total dose injected in humans was 37.8 mg/kg according to the formula for converting drug doses between humans and animals, rats demonstrated abnormal mechanical nociception manifestations. When the single injection dose of cisplatin was 2 mg/kg, the cumulative dose was 12 mg/kg, and the total injected dose in the human body was 75.6 mg/kg (based on the formula of drug dose conversion between humans and animals), rats exhibited nociceptive hypersensitivity.^8^ Mechanical pain threshold test; mechanical nociceptive hypersensitivity, hot nociceptive hypersensitivity, and cold nociceptive hypersensitivity experiments; and motor deficit tests can be used to assess the pain behavioral changes in rats and determine the successful development of the cancer pain model.^16^ Sprague–Dawley (SD) and Wistar rats are mostly selected; certain researchers have reported gender differences in the performance of rats to CIPNP, with males exhibiting more pronounced weight loss, longer latency of paw‐shrinking response, and slower motor nerve conduction, whereas females are more pronounced to certain specific histological changes.^45^

**TABLE 2:** CIPNP model of rats.

Modeling methods	Injection methods	Accumulated drug dosage (mg/kg)	Pain behavior manifestation	Rat species	Safety	Applicable research areas
0.1 mg/kg, injected every 3 days for 15 days^17^	i.p.	0.5	+MA	Wistar female rats	Deathless description	Mechanism research
0.1, 0.5, 1 mg/kg, injected once a day, thrice in total^18^	i.p.	0.3, 1.5, 3	+HH, +MH	SD male rats	Deathless description	Mechanism research
1, 2 mg/kg, injected once a week for 5 weeks^19^	i.p.	5, 10	+MA	Wistar male rats	Clearly documents that no rat deaths are recorded	Curative effect research
A. 1 mg/kg, injected once a week for 5 weeks^20^ B. 2 mg/kg, injected once a week for 4 weeks^20^	i.p.	A. 5 B. 8	+MA, –HH	Wistar male rats	Deathless description	Curative effect research
2 mg/kg, injected once^44^	i.v.	2	+HH, +MH, –CA	SD male rats	Deathless description	Developmental research
2 mg/kg, injected once a day, four times in total^5^	i.p.	8	+MA	SD male rats	Deathless description	Mechanism research
2 mg/kg, injected once a day, four times in total^6^	i.p.	8	+MA	SD male rats	Deathless description	Curative effect research
2 mg/kg, injected once every other day, four times in total^21^	i.p.	8	+MA	Wistar male rats	Deathless description	Curative effect research
2 mg/kg, injected twice a week for 4 weeks^7^	i.p.	16	+HH, +MA, +CA	SD male rats	Deathless description	Curative effect research
2 mg/kg, injected twice a week for 4.5 weeks^4^	i.p.	18	+CA, +HHO, +MA, +motor deficit	Wistar male rats	Clearly documents that no rat deaths are recorded	Curative effect research
2 mg/kg, injected twice a week for 5 weeks^8^	i.p.	20	+HH, +MA, +CA	SD male rats	Deathless description	Mechanism research
3 mg/kg, injected once a week for 3 weeks^9^	i.p.	9	+CA, +MA, –HH	SD male rats	Deathless description	Curative effect research
3 mg/kg, injected once a week for 4 weeks^10^	i.p.	12	+HHO, +MA, +motor deficit	SD male rats	Deathless description	Curative effect research
3 mg/kg, injected once a week for 4 weeks^11^	i.p.	12	+MA	SD male rats	Deathless description	Curative effect research
3 mg/kg, injected once a week for 4 weeks^12^	i.p.	12	+HHO, +MA, +motor deficit	SD male rats	Deathless description	Curative effect research
3 mg/kg, injected once a week for 4 weeks^13^	i.p.	12	+CA, +HHO	Wistar male rats	Clearly documents that no rat deaths are recorded	Curative effect research
3 mg/kg, injected once a week for 5 weeks^14^	i.p.	15	+HHO, +MA, +motor deficit	SD male and female rats	Deathless description	Curative effect research
3 mg/kg, injected once a week for 5 weeks^15^	i.p.	15	+HHO	Wistar male rats	Deathless description	Curative effect research
A. 3 mg/kg, injected once a week for 5 weeks^16^ B. 2 mg/kg, injected twice a week for 5 weeks^16^ C. 1 mg/kg, injected thrice a week for 5 weeks^16^	i.p.	A. 15 B. 20 C. 15	+CA, +HH, +MA, −motor deficit	SD male rats	Three rats in the 2‐mg/kg group died after 3, 10, and 15 days, respectively. One rat in the 3‐mg/kg group died 10 days after the last injection	Developmental research
3 mg/kg, injected twice a week^22^	i.p.	6	+HHO, −motor deficit	DA female rats	Deathless description	Mechanism research
7 mg/kg, injected once^23^	i.p.	7	+HHO	Wistar male rats	Deathless description	Curative effect research

Abbreviations: −, this behavior is not significant after evaluation of cisplatin administration; +, this behavior was significantly evaluated after cisplatin administration; CA, cold allodynia; CIPNP, chemotherapy‐induced peripheral neuropathic pain; DA, Dark Agouti rats; HH, heat hyperalgesia; HHO, heat hypoalgesia; i.p., intraperitoneal injection; i.v., intravenous injection; MA, mechanical allodynia; MH, mechanical hyperalgesia; SD, Sprague–Dawley.

### 2.2 Development of the mouse model

Researchers mostly use the modeling method of intraperitoneal injection of a single dose of 2.3 mg/kg once a day for 5 days, with 5 days of rest for 2 weeks^24^, ^25^, ^26^, ^27^ (Table 3). When a single injection dose of 2.3 mg/kg and the total dose of 6.9 mg/kg (according to the formula of drug dose conversion between humans and animals) of cisplatin was administered, which was converted to the total injection dose of 60.4 mg/kg in humans, the mechanical withdrawal reflex threshold (MWT) of mice was significantly reduced.^28^ C57BL/6J mice were used, and no significant gender difference was observed in the behavioral performance.

**TABLE 3:** CIPNP model of mice.

Modeling methods	Injection methods	Accumulated drug dosage (mg/kg)	Pain behavior manifestation	Mice species	Safety	Applicable research areas
1 mg/kg, injected once a day, seven times in total^29^	i.p.	7	+MH, +motor deficit	C3H/HeJ male mice	Deathless description	Developmental research
1 mg/kg, injected once a day, seven times in total^1^	i.p.	7	+MA, +HH	Albino male mice	Deathless description	Developmental research
2 mg/kg, injected once a day, thrice in total^30^	i.p.	6	+MA, +sensation deficit	WT male and female mice	Deathless description	Mechanism research
2 mg/kg, injected once per day, thrice in total^31^	i.p.	6	+MH	C57BL/6J male and female mice	Deathless description	Mechanism research
2 mg/kg, injected on days 0, 2, 4, and 6^32^	i.p.	8	+MH	C57BL/6J male mice	Deathless description	Mechanism research
2.3 mg/kg, injected once every other day, six times in total^33^	i.p.	13.8	+MA	C57BL/6J male mice	Deathless description	Curative effect research
2.3 mg/kg, injected once every other day, six times in total^34^	i.p.	13.8	+MA	C57BL/6J male and female mice	Deathless description	Developmental research
2.3 mg/kg, injected once every other day, six times in total^28^	i.p.	13.8	+MA, +CA, +motor deficit	Albino male mice	Deathless description	Mechanism research
2.3 mg/kg, injected once a day for 5 days for 2 weeks, with a 5‐day break^24^	i.p.	23	+HH, +MA, +motor deficit, –CA	C57BL/6J male mice	In the fourth week, mice died in the treatment group	Developmental research
2.3 mg/kg, injected once a day for 5 days for 2 weeks, with a 5‐day break^25^	i.p.	23	+MA	C57BL/6J male and female mice	Deathless description	Mechanism research
2.3 mg/kg, injected once a day for 5 days for 2 weeks, with a 5‐day break^26^	i.p.	23	+MA	C57BL/6J male mice	Deathless description	Curative effect research
2.3 mg/kg, injected once a day for 5 days for 2 weeks, with a 5‐day break^27^	i.p.	23	+MA, +CA, +motor deficit	C57BL/6J male mice	Deathless description	Curative effect research
2.3 mg/kg, injected once a day for 5 days, with a 9‐day break, repeated twice^35^	i.p.	23	+HH	BALB/c mice, gender not specified	Deathless description	Curative effect research
3 mg/kg, injected once a week for 4 weeks^12^	i.p.	12	+HH, +motor deficit	BALB/c male and female mice	Deathless description	Curative effect research
3 mg/kg, injected twice a week for 4 weeks^36^	i.p.	24	+HH, +MA, +CA	C57BL/6J male mice	Deathless description	Curative effect research
4 mg/kg, injected twice a week for 4 weeks^37^	i.p.	32	+MA	C57BL/6J female mice and A/J female mice	Deathless description	Developmental research
5 mg/kg, injected once a week for 4 weeks^38^	i.p.	20	+HH, +MA	Albino male and female mice	Deathless description	Curative effect research
5 mg/kg, injected once a week for 4 weeks^39^	i.p.	20	+CA, +MA	C57BL/6J mice, gender not specified	Deathless description	Mechanism research
5 mg/kg, injected once a week for 4 weeks^40^	i.p.	20	+MA	C57BL/6J male and female mice	Deathless description	Curative effect research
5 mg/kg, injected once a week for 4 weeks^41^	i.p.	20	+MA	C57BL/6J male and female mice	Clearly documents that no mice deaths are recorded	Curative effect research
5 mg/kg, injected once a week for 6 weeks^42^	i.p.	30	+CH, +MA	C57BL/6J male mice	On the 20th day, 9.09% of the mice died	Curative effect research
A. 10 mg/kg, injected once^43^ B. 2.3 mg/kg, injected once per day for 5 days for 2 weeks, with a 5‐day break^43^	A. i.v. B. i.p.	A.10 B.23	+MA	C57BL/6J male mice	Deathless description	Curative effect research

Abbreviations: −, this behavior is not significant after evaluation of cisplatin administration; +, this behavior was significantly evaluated after cisplatin administration; CA, cold allodynia; CH, cold hyperalgesia; CIPNP, chemotherapy‐induced peripheral neuropathic pain; HH, heat hyperalgesia; HHO, heat hypoalgesia; i.p., intraperitoneal injection; i.v., intravenous injection; MA, mechanical allodynia; MH, mechanical hyperalgesia; WT, wild type mice.

## 3 MOLDING INSPECTION METHODS

Symptoms such as abnormal sensation in the hands and feet induced by cisplatin chemotherapy in patients with CIPNP are manifested as poor mental status, slow activity, and loss of appetite in rat and mouse CIPNP models. Continuous cisplatin administration causes fur roughness and emaciation. The successful development of the CIPNP model in rats and mice is assessed by mechanical nociception, hot and cold nociception, and motor deficit tests.

### 3.1 Mechanical pain threshold testing

#### 3.1.1 Von Frey pain measurement experiment

Von Frey, also known as ciliated mechanical stimulation needles, is an alternative marker of abnormal pain in peripheral‐ and central‐sensitized states. It is the most commonly used method of mode‐forming detection. The skin or mucosal surfaces of animals were stimulated with fine wires of different diameters and stiffness (0.6, 1.0, 1.4, 2.0, 4.0, 6.0, 8.0, 10.0, 15.0, 26.0, 60.0, 100.0, 180.0, and 300.0 g), and their thresholds for responding to mechanical pain stimuli were measured. The mice were placed on a wire mesh. When mice were calm, a Von Frey filament was applied through the mesh panel to the plantar surface of their hind paws, applying enough force to bend the filament and hold the contact for 1–2 s. If the mouse moved, retracted its hind paw, or licked its foot, this was noted as an “X,” and the next lowest filament was tested. If no withdrawal response was observed, the next highest filament was evaluated and recorded as “O.” The mechanical withdrawal threshold was defined as the smallest specification of filaments eliciting the withdrawal reflex. The mechanical foot‐retraction response threshold, MWT, was recorded.^8^, ^27^ The frequency of the defibrillation response of mice was measured (in %).^46^

#### 3.1.2 Dynamic foot tester

The Ugo Basile Dynamic Plantar Tester was used, and mice were placed on a wire mesh in a transparent plastic box to acclimatize them to the environment until they were no longer exploring. The stimulation device was placed under the mouse's hind paw, and a calibrated wire was placed under the target area of the hind paw. If the start button is pressed, the device will automatically lift the wire to contact the mouse's hind paw and apply a continuous vertical force of 0–5 g at 10‐s intervals until the mouse's hind paw retracted to trigger a stop signal. The instrument automatically recorded the weight intensity threshold that triggered paw retraction.^24^

#### 3.1.3 Randall–Selitto test

The Randall–Selitto test was conducted in a quiet, temperature‐controlled room at 20–22°C from 10:00 a.m. to 4:00 p.m. The rats were acclimatized to the environment by having them climb a cylindrical restrainer with an angled vent on the side of the hind leg for 10–15 min. The instantaneous maximum pressure (in grams) during the leg‐retracting response was read using a Basile Algesy meter as the threshold of an injurious sensation. It was measured using a hand‐held instrument; the pressure was increased until the rat underwent withdrawal or vocalization. Measurements were recorded at 5‐min intervals, and the average of three measurements was defined as the paw pressure pain threshold.^44^

### 3.2 Cold nociceptive hypersensitivity test

#### 3.2.1 Acetone test

Rats were placed in individual plastic cages on an overhead wire mesh and allowed to acclimatize for at least 15 min until exploratory behavior was no longer observed. Acetone was filled in a 1‐mL syringe without a needle tip. Air bubbles were removed from the syringe before using acetone. A drop of acetone (~20 μL) was applied to the plantar surface of the rat's hind paw through a wire mesh, avoiding contact between the syringe and the paw. And the time of the rat's paw‐retraction reaction was recorded for 60 s after acetone application. The paw‐retraction reaction was manifested as rapid paw fluttering, trembling, paw biting, or paw licking. Paws were evaluated alternately left and right until five measurements were obtained for each paw, with an interval of ~3 min between the left‐ and right‐paw tests. Cold anomalous pain tests were performed on days 0, 4, 8, 12, and 16.^9^, ^39^, ^42^

#### 3.2.2 Cold water bath rat tail withdrawal method

The rat's tail was placed in a 10°C water bath; the time the tail was immersed in the cold water bath was recorded, with an interval of 5 min between each measurement. The average of three measurements was taken, with a cutoff time of 15 s.^44^

#### 3.2.3 Cold plate method

The cold plate device consists of a transparent glass cylinder and a cold plate. The temperature of the metal plate was set to −5 to 4°C, and mice were placed in the test area for at least 30–60 min for acclimatization. The reaction time of mice to cold stimuli, such as paw licking and jumping, was observed to assess the sensitivity of mice to cold nociception.^24^

### 3.3 Thermal nociceptive hypersensitivity testing

#### 3.3.1 Thermal radiation method

##### Hargreaves test

The rat was placed in an 18 × 8 × 8‐cm transparent plastic box with a piece of glass at the bottom that could transmit infrared rays. After the rats had adapted to the environment for 30–60 min, 50 W of radiant heat was projected through an elliptical aperture onto the heel of the rat's hind paw. The beam was automatically switched off when the rat lifted its paw. The time from the emission of the thermal stimulus to the time when the mouse lifted or licked the hind paw, that is, thermal stimulus response latency, was observed and recorded. Each hind paw was measured thrice, each stimulus interval was 5 min, and the average of the three measurements was taken.^18^, ^44^

##### Thermal radiation tail‐flick method

In this method, radiation was applied to the distal one third of the rat's tail using an 8 V/50 W‐radiation tail‐flick device (tail‐flick device, May Tic., Ankara, Turkey). The thermal stimulus was interrupted when the tail moved, and the latency of the tail flick was recorded. The cutoff time was set to 10 s to prevent tissue damage to the tail by thermal radiation.^15^

#### 3.3.2 Hot plate test method

Mice were placed in the test area and adapted to the environment for 30–60 min. The temperature of the hot plate tester (Columbus) was set to 55°C to measure the latency of the hot plate response for 60 min before and after the administration of the drug to mice. The cutoff time was set to 30 s to avoid tissue damage to mice.^39^

#### 3.3.3 Hot water bath mouse tail immersion experiment

Each mouse was wrapped in a wet paper towel, except for the tail, three to four times, each time lasting 1 min. Then, one third of the distal end of the mouse tail was immersed in a water bath after the mice had adapted to the test environment. The temperature of the water bath was preset at 50.5C ± 0.5°C, and the cutoff time was set to 20 s. The next immersion was performed when the tails of mice were relaxed. The test was restarted if the mice were agitated. The latency of violent tail flicking was recorded, with an interval of at least 30 min between each time, and the average of three measurements was taken.^24^

### 3.4 Motor function tests

#### 3.4.1 Rotary rod test

The rotarod test is a method to assess the motor function of rats for motor coordination and balance. The mice were placed on an accelerated rotating rod, whose speed was increased from 4 to 40 rpm in 300 s. A stopwatch was used to record the endurance latency of mice to fall from the rotating rod. Mice were acclimatized for 4 days before the experiment, with 70 s per session on day 1, gradually increasing to 300 s per session on day 4. In case of any premature falls during training, mice were gently returned to the rotating bar. On day 4, the mice were divided into different groups (*n* = 8 per group), and the time of dropping the rod and the speed of the rotating rod at the time of dropping were recorded. The average time on the rotating rod before and after drug administration was calculated to assess their motor coordination and balance ability.^14^ Better balance and coordination of mouse limbs resulted in their longer stay on the rotarod, whereas worse balance and coordination resulted in shorter durations.^29^

#### 3.4.2 Adhesive removal test

The adhesive removal test was used to assess murine sensorimotor dysfunction and motor asymmetry. We placed the mice in a 10 × 20 × 10‐cm transparent plastic box, after which two similar tapes were applied to the hairless portion of each of their front paws using the same pressure. The time between the first contact with the tape and the complete removal of the tape was recorded.^30^

#### 3.4.3 Grip strength test

A grip strength meter (Stoelting Wood Dale, IL), consisting of a force sensor with a digital display and a metal plate with a hanger, was used. Each mouse was placed on the Stoelting Wood Dale plate. The mouse grasped the probe firmly with its forelimbs, and its tail was pulled with a gradually increasing force until it could not grasp the hanger. Ensuring that the mouse grasped the top of the grid and the trunk remained horizontal, the maximum grip strength value of the mouse displayed on the screen was recorded and averaged thrice.^24^

## 4 DISCUSSION

The latest statistics from the International Agency for Research on Cancer estimated nearly 20 million new cancer cases in 2022, with lung cancer as the most common cancer (200 000 new cases) accounting for 12.4% of the new cancer cases worldwide.^47^ The National Comprehensive Cancer Network in its 2024 guidelines for non‐small‐cell lung cancer included ositinib + pemetrexed + cisplatin or carboplatin as the first‐line treatment regimen for nonsquamous cell carcinoma. Initially four cycles of systemic therapy with cisplatin are recommended, and continuation for six cycles may be considered if the patient tolerates the treatment well.^48^ It has been reported that 30%–40% of patients treated with cisplatin develop distal limb pain and sensory abnormalities, which can progress to severe neuropathic pain and sensory ataxia in advanced stages.^24^ Furthermore, 47% of patients continue to have symptoms of CIPNP at 6 years after the end of chemotherapy and even throughout life. The mechanism of CIPNP is intricate. Studies have demonstrated that it is intricately related to chemotherapeutic drug–induced intraepidermal nerve fiber damage, ion channel dysregulation, cytokine upregulation, and immune activation of the nervous system.^49^ No drugs with preventive and curative effects are available, and the 2014 ASCO guidelines recommend duloxetine analgesic treatment for severe pain. Moreover, we lack established diagnostic criteria for CIPNP, and more clinical trials and animal studies are urgently required to address this major clinical problem with regard to the preventive and curative drugs for different types of chemotherapeutic agents and their induced CIPNP.

Researchers generally select mechanical nociception, cold nociception, and hot nociception experiments to determine whether the cancer pain model is successful; however, the detection standard of pain behavior tests is still controversial. Among them, the mechanical pain test is the most commonly used modeling test, and the Von Frey method is regarded as the “gold standard” of mechanical pain tests because of its classical, simple, and efficient nature. However, it has disadvantages such as low accuracy and poor reproducibility. Therefore, researchers have recently used electronic mechanical pain testers to improve their accuracy.^24^ Cold pain threshold and heat pain threshold detection are also commonly used for mold‐forming detection; researchers mostly use hot and cold plate pain testers for assessment, which has the advantages of precise temperature control, rapid cooling, and prevention of scalded animals. The CIPNP model is mostly used in three types of medical research: mechanism research, efficacy research, and developmental research. Mechanistic research refers to the study of the pathogenesis of cisplatin‐induced CIPNP and the mechanism of drug action in animal models and their pathological mechanisms. In the CIPNP mechanism study model, one to two kinds of behavioral tests are evaluated using SD rats (1–2 mg/kg, injected twice a week for 5 weeks) or C57BL/6J mice (2.3 mg/kg, injected once a day for 5 days, resting for 5 days, for 2 weeks).^8^, ^25^ Efficacy studies are primarily used to evaluate the effectiveness of a drug or compound in treating CIPNP. The accuracy and reliability of animal model experiments are crucial to the efficacious evaluation and success of the drug development process. In the efficacy study model, multiple pain behavior detection criteria should be selected to obtain a comprehensive assessment, and a scientifically rich animal model evaluation system should be used. SD rats (3 mg/kg, injected once a week for 4 weeks) or C57BL/6J mice (2.3 mg/kg, injected once a day for 5 days, with 5 days of rest for 2 weeks; or a high dose of 5 mg/kg, injected once a week for 4 weeks) can be selected.^10^, ^11^, ^12^, ^13^, ^26^, ^27^ Developmental studies are based on the results of applied research and current science and technology to promote the development of CIPNP research. Developmental studies can select SD rats (2 mg/kg, injected twice a week for 5 weeks) or C57BL/6J mice (2.3 mg/kg, injected every day for six times of modeling).^16^, ^34^ Limitations of animal model experiments should be acknowledged. These include species differences between animals and humans, and the experimental results may be affected by several factors. It is necessary to fully consider the limitations and implement the corresponding measures to reduce the error and improve the accuracy of the experiment when conducting animal model experiments. Domestic and foreign researchers have observed the model formation test for no more than 2 weeks (14 days), and the time point of the behavioral test is primarily every 5 days or once a week; however, certain studies found that the animals exhibited nociceptive allergy at the beginning of the first week and severe nociceptive allergy during the second and third weeks.^50^ Therefore, it is recommended that researchers increase the observation time of behavioral tests to better develop a high‐quality model.

The 2014 ASCO guidelines recommend only duloxetine as a first‐line agent for the treatment of CIPNP, whereas the German Society of Neurology guidelines recommend capsaicin patches as a second‐line agent for treating any kind of neuropathic pain.^51^, ^52^ However, the treatment of CIPNP is not yet supported by high‐quality studies and lacks validation in randomized clinical trials. In this regard, the application of rat and mouse models of CIPNP can promote research on the pathogenesis and prevention strategies of CIPNP, and thereby improve the quality of life of patients and their adherence to treatment regimens.

## AUTHOR CONTRIBUTIONS

**Ningxin Li:** Conceptualization; methodology; writing – original draft. **Mingzhu Li:** Funding acquisition; supervision; writing – review and editing. **Shengbo Jin:** Resources; validation; visualization. **Jun Yu:** Conceptualization; investigation; methodology. **Hongzhe Wei:** Data curation; validation. **Wenping Wang:** Conceptualization; supervision; validation. **Siyao Ma:** Investigation. **Yuxin Jiang:** Formal analysis; software. **Qian Liu:** Methodology. **Huini Yao:** Conceptualization.

## FUNDING INFORMATION

This study was funded by the National Natural Science Foundation of China (grant number 82104838), the China Health Promotion Foundation Spark Program (grant number XH‐D001), and the Liaoning Provincial Key Research and Development Program (grant numbers 2024JH2/102500062).

## CONFLICT OF INTEREST STATEMENT

The authors have no relevant financial or nonfinancial interests to disclose.

## ETHICS STATEMENT

This review article was not subject to any ethics approval from the institutions.

## CONSENT TO PARTICIPATE

All authors provided their consent to participate.

## CONSENT FOR PUBLICATION

All authors gave their consent for publication of this review article.

## Data Availability

No data was used for the research described in the article.
